# A shared vascular-hypoxic transcriptional program is associated with immune organization in glioblastoma

**DOI:** 10.3389/fimmu.2026.1960638

**Published:** 2026-10-28

**Authors:** Kinga Jaskuła, Adrian Szczepaniak, Clara Kugler, Sean E. Lawler

**Affiliations:** 1 Department of Experimental Genomics, Institute of Genetics and Animal Biotechnology, Polish Academy of Sciences, Jastrzębiec, Poland; 2 Department of Pathology and Laboratory Medicine, Legorreta Cancer Center, The Warren Alpert Medical School, Brown University, Providence, RI, United States; 3 Department of NeuroOncology, Mossakowski Medical Research Institute, Polish Academy of Sciences, Warsaw, Poland; 4 Department of Pathology and Laboratory Medicine, Legorreta Cancer Center, Brown University, Providence, RI, United States; 5 Doctoral School of Translational Medicine, Centre of Postgraduate Medical Education, Warsaw, Poland

**Keywords:** angiogenesis, glioblastoma, hypoxia, immune cells, vascular niche

## Abstract

Glioblastoma (GBM) contains spatially distinct vascular and hypoxia/necrosis-associated compartments, but the extent to which these classical anatomical regions share transcriptional programs linking vascular remodeling with immune-related processes remains incompletely defined. Here, Ivy Glioblastoma Atlas Project (IvyGAP) anatomical transcriptomic data were used as a discovery framework and integrated with independent bulk and single-cell transcriptomic resources to characterize the cellular, immune and clinical context of a shared vascular-hypoxic program. Analysis of regionally defined IvyGAP compartments identified 1,114 genes enriched in vascular-associated regions and 403 genes enriched in hypoxia/necrosis-associated regions, with 95 genes shared between the two comparisons. Functional enrichment implicated angiogenesis, extracellular matrix remodeling, inflammatory signaling, chemotaxis, complement/coagulation and cytokine-related processes. The shared signature was further resolved into angiogenesis and vascular remodeling, immune and inflammatory signaling, and shared immunovascular gene subsets. Single-cell mapping showed expression of these programs across endothelial, perivascular and myeloid populations, with immune/inflammatory components also detectable in vascular-associated cell populations. Vascular remodeling genes were positively associated with inflammatory genes, stromal and exclusion scores, myeloid-enriched immune populations and immunoregulatory markers. Representative genes from each functional subset showed preferential expression in mesenchymal GBM across TCGA and CGGA, while survival modeling using the predefined combined immunovascular program separated patients into groups with distinct overall survival across three GBM cohorts. Together, these findings identify an anatomically anchored vascular–hypoxic transcriptional program associated with vascular remodeling, inflammatory signaling, immune-context features and adverse clinical outcome, providing a cross-dataset framework for interpreting immunovascular relationships in GBM.

## 1 Introduction

Glioblastoma (GBM) is characterized by marked spatial heterogeneity, with vascular remodeling, hypoxia, necrosis and immune dysfunction distributed unevenly across the tumor. This heterogeneity is reflected in densely cellular regions, abnormal vascular structures, hypoxic and necrotic areas, and invasive margins that form anatomically distinct yet biologically interconnected compartments (1–3). Microvascular proliferation and necrosis, frequently accompanied by pseudopalisading tumor cells, are central histopathological hallmarks of GBM and reflect the close relationship between vascular dysfunction, oxygen deprivation, and tumor adaptation (4, 5). Recent spatial profiling studies have further shown that these anatomical regions represent multicellular microenvironments with distinct cellular and transcriptional states (2, 3, 6).

This spatial heterogeneity is particularly relevant to the GBM immune microenvironment. GBM is dominated by myeloid populations, including brain-resident microglia and infiltrating monocyte-derived macrophages, whereas lymphocytes are generally less abundant (7–9). Immune-cell composition varies across anatomical regions: blood-derived macrophages are enriched in perivascular and necrotic regions, while hypoxic niches can sequester and functionally reprogram tumor-associated macrophages and cytotoxic T cells (10–12). Thus, immune dysfunction in GBM occurs within a heterogeneous tissue environment in which vascular abnormalities, hypoxia, extracellular matrix remodeling and inflammatory signaling vary across anatomical regions.

Vascular and hypoxia/necrosis-associated compartments are of particular interest within this architecture. Although biologically interconnected, these compartments have often been studied through distinct biological frameworks: vascular regions primarily as sites of angiogenesis and barrier dysfunction, and hypoxic or perinecrotic regions in the context of metabolic stress, treatment resistance and immunosuppressive signaling. Recent mechanistic studies increasingly indicate reciprocal relationships between these compartments and the immune microenvironment. GBM-associated endothelial cells can promote immunoregulatory macrophage states through angiocrine signaling, whereas hypoxia-associated macrophage populations can alter endothelial integrity and vascular permeability (12–14). Spatial studies have further identified perivascular microenvironments containing endothelial, stromal, myeloid and malignant populations (3, 15). Together, these observations suggest that vascular remodeling, hypoxic stress and immune-related processes represent closely associated components of the GBM microenvironment.

Previous studies have therefore resolved complementary aspects of GBM spatial biology. Ivy Glioblastoma Atlas Project (IvyGAP) established transcriptional programs associated with classical histopathological structures (1), while subsequent single-cell and spatial studies have defined malignant, myeloid, endothelial and perivascular cell states and their spatial relationships (2, 3, 14, 15). What remains unresolved is whether vascular and hypoxia/necrosis-associated GBM compartments share a defined transcriptional program that integrates vascular-remodeling and immune-related features. The functional organization of such a program and its relationships with cellular, immune and clinical features across independent datasets also remain incompletely characterized.

Resolving this question is important because a transcriptional program spanning histologically distinct niches could reveal a shared microenvironmental state that is not captured by compartment-specific analyses and could provide a framework for linking vascular remodeling to immune context and clinical behavior. Accordingly, IvyGAP anatomical transcriptomic data were used as a discovery framework to determine whether vascular-associated and hypoxia/necrosis-associated GBM compartments share a common transcriptional program containing both vascular-remodeling and immune-related components. The resulting vascular-hypoxic signature was then examined across independent bulk and single-cell datasets to define its cell-type expression patterns, immune-context associations, relationship to GBM transcriptional subtype, and association with patient outcome. By integrating anatomically defined compartments with cellular, immune and clinical transcriptomic contexts, this study identifies a shared vascular-hypoxic program that extends previous descriptions of individual GBM niches and provides a transcriptional framework for interpreting immunovascular relationships in GBM.

## 2 Materials and methods

This study used an integrative anatomical transcriptomics approach to identify a transcriptional program shared by vascular- and hypoxia/necrosis-associated GBM compartments and to examine its cellular, immune and clinical context across independent datasets. Regionally annotated IvyGAP data were used for signature discovery, followed by functional characterization, single-cell mapping, immune-context analyses, GBM subtype profiling and survival analysis using publicly available datasets and analytical platforms.

### 2.1 Identification and functional characterization of the vascular-hypoxic signature

Regionally annotated RNA-seq data were obtained from IvyGAP. Hyperplastic blood vessels and microvascular proliferation (HBV/MVP) were analyzed as vascular-associated compartments, whereas perinecrotic zone and pseudopalisading cells around necrosis (PNZ/PAN) were analyzed as hypoxia/necrosis-associated compartments. Leading edge, infiltrating tumor and cellular tumor regions (LE/IT/CT) served as reference compartments. Differential expression was assessed separately for HBV/MVP versus LE/IT/CT and PNZ/PAN versus LE/IT/CT, with enriched genes defined by fold-change > 2 and p < 0.05. The intersection of the two gene sets defined the shared vascular-hypoxic signature. For anatomical heatmaps, repeated samples from the same donor and anatomical region were averaged so that each donor contributed at most one expression value per region.

Functional enrichment analysis was performed for vascular-associated genes, hypoxia/necrosis-associated genes and the shared vascular-hypoxic signature using Gene Ontology Biological Process and KEGG pathway annotations (ShinyGO v0.80) (16). Enriched terms were ranked by FDR-adjusted significance, and terms with FDR < 0.05 were considered significant. Protein functional-association analysis of the shared vascular–hypoxic signature was performed using STRING database (17). The network was generated using a high-confidence minimum required interaction score of 0.700 and Markov Cluster Algorithm clustering.

To resolve the biological structure of the 95-gene shared vascular-hypoxic signature, genes were assigned to focused component gene programs based on their mapping to enriched functional annotations. Genes annotated to GO:0001568 blood vessel development, GO:0048514 blood vessel morphogenesis, GO:0001944 vasculature development, GO:0001525 angiogenesis, GO:0009611 response to wounding, KEGG focal adhesion, KEGG ECM-receptor interaction, or related tissue-remodeling pathways were classified as angiogenesis and vascular remodeling genes. Genes annotated to GO:0006954 inflammatory response, GO:0006935 chemotaxis, GO:0002376 immune system process, leukocyte and myeloid-cell migration terms, KEGG IL-17 signaling, KEGG TNF signaling, KEGG NF-κB signaling, KEGG cytokine-cytokine receptor interaction, KEGG chemokine signaling, KEGG complement and coagulation cascades were classified as immune and inflammatory signaling genes. Genes annotated to both vascular/tissue-remodeling and immune/inflammatory categories were classified as the shared immunovascular genes. Genes from the 95-gene signature that did not meet criteria for these focused panels were retained in the complete shared vascular–hypoxic signature and are reported in **Supplementary File 1**. These genes were not included in the primary subset-level analyses when their annotations were broader, or less directly aligned with the vascular-immune focus of the study.

### 2.2 Cross-dataset cellular, molecular and immune-context analyses

GBM subtype-associated expression patterns were assessed using GlioVis (18), with TCGA-GBM RNA-seq used as the primary cohort and CGGA as an independent validation cohort. Highly enriched genes from each functional program were compared across classical, mesenchymal and proneural transcriptional subtypes using platform-implemented statistical testing.

Single-cell expression patterns of the component gene programs were assessed using publicly available UCSC Cell Browser datasets (19) generated from GBM-associated and brain vascular single-cell transcriptomic resources (20). A GBM single-cell dataset enriched for endothelial and perivascular populations was used to map the three functional gene programs across vascular, stromal, immune and malignant populations, and an independent brain vascular reference dataset was used to assess their distribution across annotated vascular and perivascular cell types. Pre-existing cell annotations and low-dimensional embeddings were used for visualization. Dot plots summarized average expression and the fraction of cells with detectable expression within each population.

Immune-context associations were assessed using TIMER3 (21) and GSCA (22). Spearman correlations were calculated between angiogenesis/vascular-remodeling genes and immune/inflammatory genes, immune, stromal and exclusion scores, and inferred immune-cell populations. Associations with a predefined panel of checkpoint/T-cell dysfunction, immunosuppressive cytokine-signaling and myeloid immunoregulatory markers were also evaluated.

To assess associations with immunoregulatory features, three predefined, literature-informed marker panels were used: checkpoint/T-cell dysfunction (CD274, PDCD1LG2, HAVCR2, LAG3, TIGIT, CTLA4), immunosuppressive cytokine signaling (TGFB1, TGFB2, IL10), and myeloid immunoregulation (CSF1R, CD163, MRC1, MSR1, LGALS9, IDO1). The panels comprised commonly used markers of these immunoregulatory processes and were defined independently of the IvyGAP-derived vascular-hypoxic signature. Single-cell expression was assessed using the original cell-type annotations from the source dataset. For TCGA-GBM, gene expression values were z-score standardized and averaged within each panel to generate composite signature scores, which were compared across classical, mesenchymal, and proneural subtypes. Associations with EGFR copy-number status were evaluated in TCGA-GBM using the Firehose Legacy dataset accessed through cBioPortal (23).

### 2.3 Clinical association and statistical analysis

Survival analyses were performed using SurvExpress (24) and GEPIA2 analyzer (25). The vascular-hypoxic gene program, comprising angiogenesis and vascular remodeling genes, immune and inflammatory signaling genes, and shared immunovascular genes, was submitted as a predefined multigene signature. Patients were stratified into low- and high-risk groups using the platform-implemented Cox proportional hazards model and prognostic index. Hazard ratios, confidence intervals and p-values were extracted from SurvExpress outputs. Subtype-stratified overall survival analyses of the three functional gene subsets were performed in TCGA-GBM using GEPIA2. Classical, mesenchymal, and proneural tumors were analyzed separately, and patients within each subtype were stratified into low- and high-signature groups using the predefined GEPIA2 cutoff. Kaplan–Meier curves, hazard ratios, and log-rank p-values were obtained from the platform output.

Unless otherwise specified, statistical analyses followed the procedures implemented in the respective public platforms. Differential expression analyses used nominal (unadjusted) p-values, whereas functional enrichment analyses were evaluated using FDR-adjusted p-values. Correlation analyses are reported as Spearman correlation coefficients with multiple-testing-adjusted p-values obtained from the respective analytical platforms. SurvExpress analyses report hazard ratios with confidence intervals and platform-derived p-values, whereas subtype-stratified GEPIA2 survival analyses report hazard ratios and log-rank p-values. No additional multivariable adjustment for clinical or molecular confounders was performed beyond the models implemented by the respective source platforms. Heatmaps were generated using Morpheus, whereas additional statistical analyses and graphical outputs were performed using GraphPad Prism 10. Statistical thresholds and visualization parameters are reported in the corresponding figure legends.

## 3 Results

### 3.1 IvyGAP identifies a shared vascular-hypoxic transcriptional program enriched for remodeling and immune-related processes

To determine whether vascular- and hypoxia/necrosis-associated GBM compartments share common transcriptional features, regionally annotated IvyGAP data were analyzed. Hyperplastic blood vessel and microvascular proliferation regions (HBV/MVP) were compared with reference tumor compartments comprising leading edge, infiltrating tumor and cellular tumor regions (LE/IT/CT), identifying 1,114 genes enriched in vascular-associated regions (**Figure 1A**; **Supplementary File 1**). In parallel, perinecrotic zone and pseudopalisading cells around necrosis (PNZ/PAN) were compared with the same reference compartments, identifying 403 genes enriched in hypoxia/necrosis-associated regions (**Figure 1B****;** **Supplementary File 1**). The intersection of these two gene sets comprised 95 genes and was defined as the shared vascular-hypoxic signature (**Figure 1C**; **Supplementary File 1**). Projection of this signature across IvyGAP anatomical structures confirmed preferential expression in vascular- and hypoxia/necrosis-associated compartments relative to LE, IT and CT regions (**Figure 1D**).

**Figure 1:**
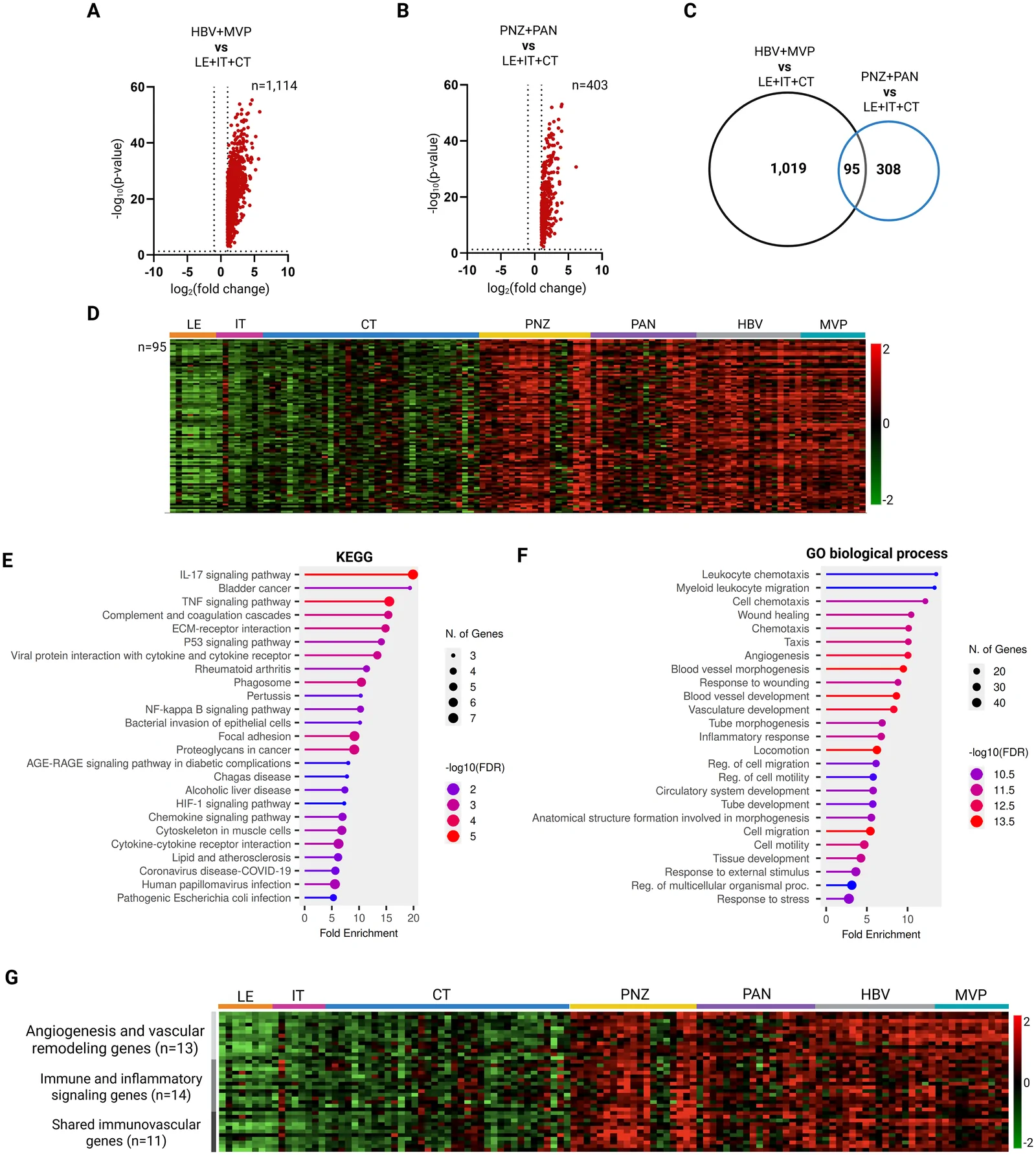
IvyGAP identifies a shared vascular-hypoxic transcriptional program enriched for remodeling and immune-related processes. Regionally annotated transcriptomic data from the IvyGAP were analyzed to identify genes enriched in vascular- and hypoxia-associated anatomical compartments of GBM. **(A)** Volcano plot showing genes enriched in vascular-associated regions, defined as hyperplastic blood vessels and microvascular proliferation (HBV/MVP), compared with reference tumor compartments comprising leading edge, infiltrating tumor and cellular tumor regions (LE/IT/CT). This comparison identified 1,114 enriched genes. The y-axis represents −log10(unadjusted p-value). **(B)** Volcano plot showing genes enriched in hypoxia/necrosis-associated regions, defined as perinecrotic zone and pseudopalisading cells around necrosis (PNZ/PAN), compared with the same reference tumor compartments (LE/IT/CT). This comparison identified 403 enriched genes. The y-axis represents −log10(unadjusted p-value). **(C)** Venn diagram showing the overlap between genes enriched in the vascular-associated and hypoxia/necrosis-associated comparisons. The 95 commonly enriched genes were defined as the shared vascular-hypoxic signature. **(D)** Heatmap showing scaled expression of the 95-gene shared vascular–hypoxic signature across IvyGAP anatomical regions, including LE, IT, CT, PNZ, PAN, HBV and MVP. **(E)** KEGG pathway enrichment analysis of the 95 shared genes. Dot size indicates the number of genes assigned to each pathway, and color indicates statistical significance expressed as −log_10_(FDR). **(F)** Gene Ontology biological process enrichment analysis of the 95 shared genes. Dot size indicates the number of genes assigned to each term, and color indicates statistical significance expressed as −log_10_(FDR). **(G)** Heatmap showing scaled expression of three component gene programs within the 95-gene shared vascular-hypoxic signature: angiogenesis and vascular remodeling genes, immune and inflammatory signaling genes, and shared immunovascular genes. Genes included in each component program are listed in **Supplementary Table S1**.

Functional enrichment analyses indicated that the shared vascular-hypoxic signature encompassed both tissue-remodeling and immune-related processes. Vascular-associated genes were strongly enriched for angiogenesis and vessel-related biological processes, whereas hypoxia/necrosis-associated genes showed enrichment of vasculature-related terms together with immune and inflammatory processes (**Supplementary Figures S1A, B**). Within the 95-gene shared signature, STRING analysis identified functional modules associated with extracellular matrix interactions, collagen binding, integrin-mediated cell-surface interactions, neutrophil chemotaxis, inflammatory signaling and TLR4-related responses (**Supplementary Figure S1C**). Consistently, KEGG analysis identified enrichment of IL-17, TNF, NF-κB, cytokine-cytokine receptor interaction, chemokine signaling, complement and coagulation cascades, phagosome, ECM-receptor interaction, focal adhesion and proteoglycans in cancer pathways (**Figure 1E**). Gene Ontology Biological Process analysis showed enrichment for chemotaxis, angiogenesis, wound healing, blood vessel morphogenesis and development, inflammatory response, and regulation of cell migration and motility (**Figure 1F**).

To further resolve the biological composition of the shared vascular-hypoxic signature, the 95 genes were stratified according to enriched Gene Ontology Biological Process and KEGG pathway annotations. This analysis identified three functional gene subsets: angiogenesis and vascular remodeling genes, immune and inflammatory signaling genes, and shared immunovascular genes annotated to both vascular/tissue-remodeling and immune/inflammatory processes (**Supplementary Table S1**; **Figure 1G**). All three functional subsets showed preferential expression in HBV, MVP, PNZ and PAN relative to LE, IT and CT regions (**Figure 1G**), indicating that the shared signature captures coordinated vascular remodeling, inflammatory signaling and immunovascular transcriptional features enriched within anatomically defined vascular-hypoxic compartments.

### 3.2 Single-cell mapping and immune-context analyses associate three functional gene subsets with endothelial-perivascular compartments, myeloid populations and immunoregulatory features

To assess the cellular distribution of the three functional gene subsets within the shared vascular-hypoxic signature, expression patterns were mapped onto a publicly available GBM single-cell dataset enriched for endothelial and perivascular populations (19, 20). Angiogenesis and vascular remodeling genes showed prominent expression in endothelial and perivascular compartments, including endothelial cells, pericytes and smooth muscle cells, with additional signal in fibroblast-like populations and microglia/macrophages (**Figures 2A, B**). Immune and inflammatory signaling genes were most strongly detected in myeloid populations, particularly microglia/macrophages and neutrophils, but were also present in vascular-associated compartments, including endothelial cells, pericytes, smooth muscle cells and fibroblast-like cells (**Figures 2C, D**). Shared immunovascular genes showed broader expression across endothelial, perivascular stromal, myeloid and selected malignant or stem-like compartments (**Figures 2E, F**).

**Figure 2:**
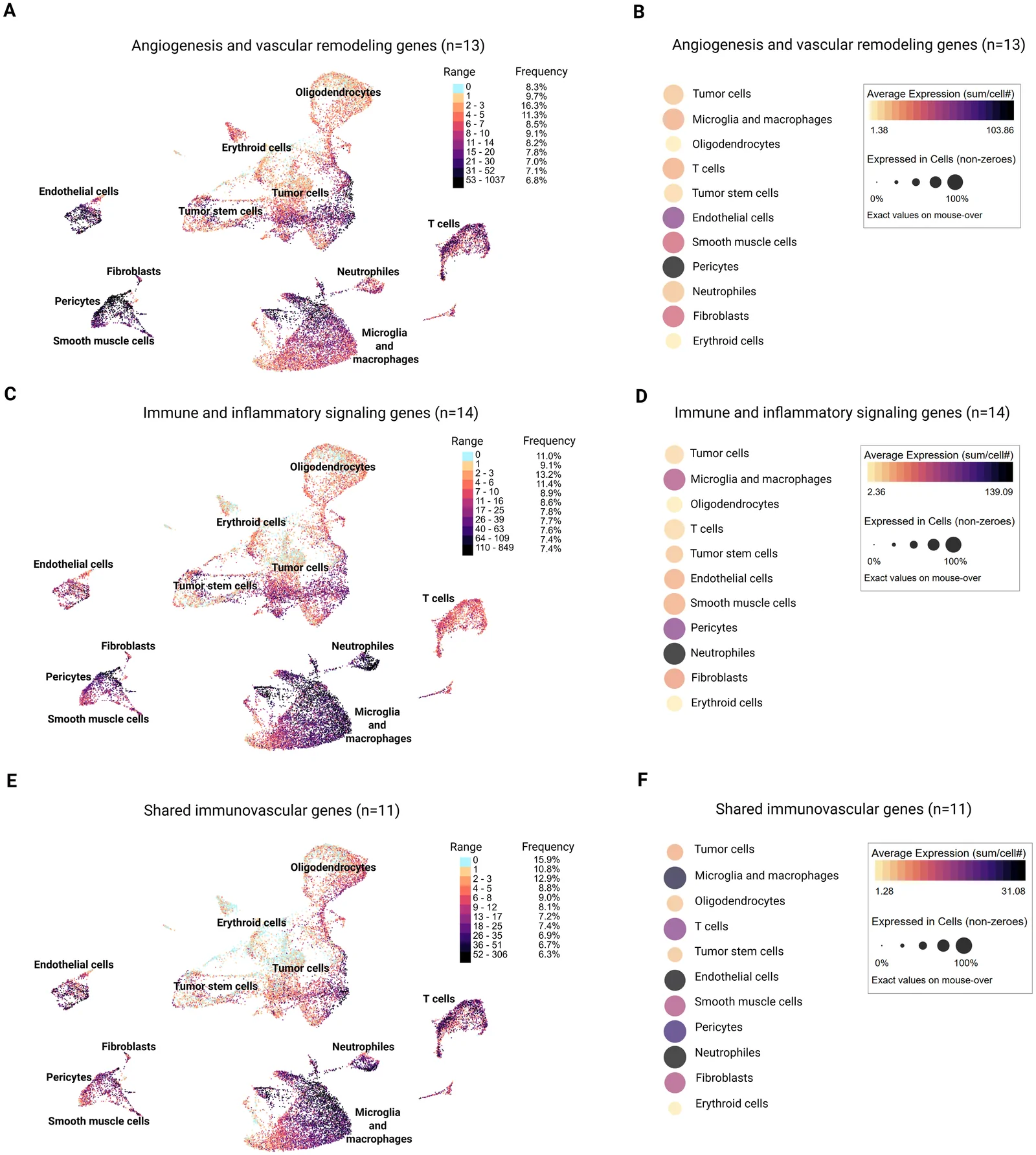
Single-cell mapping associates functional gene subsets within the shared vascular-hypoxic signature with endothelial-perivascular and myeloid compartments in GBM. **(A, C, E)** UMAP visualization of the three functional gene subsets in a publicly available GBM single-cell dataset enriched for endothelial and perivascular populations: angiogenesis and vascular remodeling genes **(A)**, immune and inflammatory signaling genes **(C)**, and shared immunovascular genes **(E)**. **(B, D, F)** Dot-plot summaries of the same three functional gene subsets across annotated cell populations: angiogenesis and vascular remodeling genes **(B)**, immune and inflammatory signaling genes **(D)**, and shared immunovascular genes **(F)**. Dot color represents average expression, whereas dot size indicates the fraction of cells with detectable expression within each population. Annotated cell populations include endothelial cells, pericytes, smooth muscle cells, fibroblasts, microglia/macrophages, neutrophils, T cells, tumor cells, tumor stem-like cells, oligodendrocytes and erythroid cells.

The same three functional gene subsets were then evaluated in an independent brain vascular single-cell reference dataset. This analysis showed a similar enrichment of angiogenesis and vascular remodeling genes in endothelial and perivascular populations, whereas immune and inflammatory signaling genes showed predominant expression in myeloid compartments (**Supplementary Figures S2A–D**). Immune and inflammatory signaling genes also remained detectable in endothelial and perivascular stromal populations, indicating that this component of the vascular-hypoxic program was not restricted to immune-cell compartments. Shared immunovascular genes showed a broad distribution across vascular, stromal, myeloid, lymphoid and selected neural-lineage populations (**Supplementary Figures S2E, F**).

Immune-context associations were subsequently assessed for angiogenesis and vascular remodeling genes. These genes showed broadly positive correlations with immune and inflammatory signaling genes, including cytokine, chemokine and myeloid-associated mediators (**Figure 3A**). Several angiogenesis and vascular remodeling genes were also positively associated with stromal and exclusion scores, whereas associations with the global immune score were more variable (**Figure 3B**). Correlation analysis with inferred immune-cell populations showed positive associations between the angiogenesis and vascular remodeling gene score and monocytes, macrophages, Th17 cells, natural killer T cells, regulatory T-cell populations and cytotoxic cells. In contrast, negative associations were observed with B cells, CD8+ naive cells and γδ T cells (**Figure 3C**).

**Figure 3:**
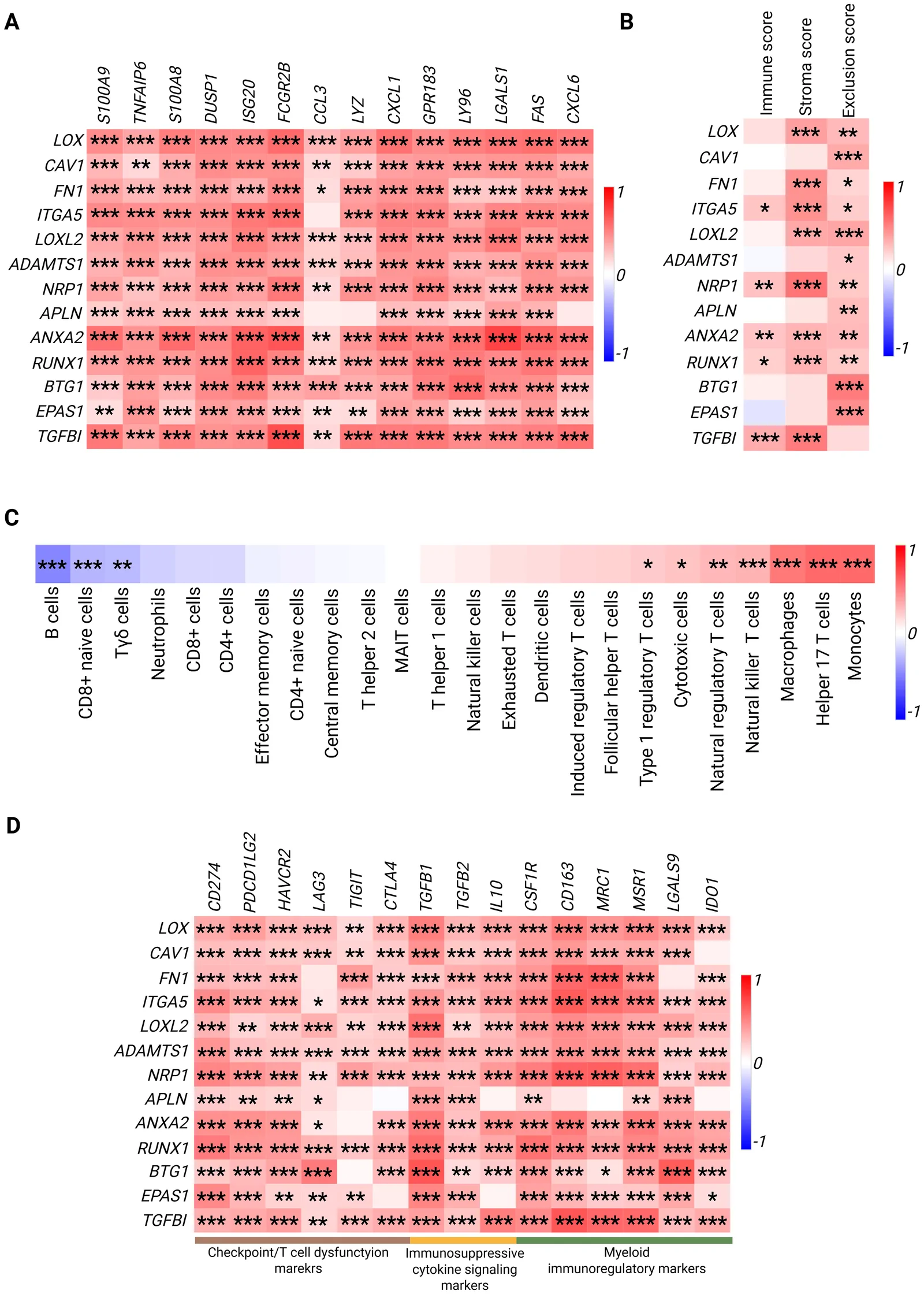
Correlation analysis links vascular remodeling genes with immune-context features in GBM. **(A)** Spearman correlation matrix showing associations between angiogenesis and vascular remodeling genes and immune/inflammatory signaling genes. Red indicates positive correlations and blue indicates negative correlations. **(B)** Correlation matrix showing associations between angiogenesis and vascular remodeling genes and immune-related, stromal and exclusion scores. Red indicates positive correlations and blue indicates negative correlations. **(C)** Correlation heatmap showing associations between the angiogenesis and vascular remodeling gene score and inferred immune-cell populations in GBM. Red indicates positive correlations and blue indicates negative correlations. **(D)** Spearman correlation matrix showing associations between angiogenesis/vascular remodeling genes and a three predefined immunoregulatory marker panels representing checkpoint/T-cell dysfunction, immunosuppressive cytokine signaling, and myeloid immunoregulation. For all panels, red indicates positive correlations and blue indicates negative correlations. Asterisks indicate statistical significance after multiple-testing correction: *adjusted p < 0.05, **adjusted p < 0.01 and ***adjusted p < 0.001. Exact correlation coefficients and adjusted *p*-values for all comparisons are provided in **Supplementary File 1**.

To further characterize immunoregulatory features associated with angiogenesis and vascular remodeling genes, these genes were correlated with a predefined marker panel comprising checkpoint/T-cell dysfunction markers, immunosuppressive cytokine signaling markers and myeloid immunoregulatory markers. Predominantly positive associations were observed across these marker classes (**Figure 3D**).

To further examine the cellular and molecular context of these immunoregulatory features, the three predefined marker classes were analyzed across GBM cell populations and molecular subtypes. Single-cell mapping showed that checkpoint/T-cell dysfunction markers were predominantly detected in microglia/macrophages and T cells, with lower expression across other cellular compartments (**Supplementary Figure S3A**). Immunosuppressive cytokine signaling markers showed a broader distribution, with prominent expression in pericytes as well as microglia/macrophages, endothelial cells and other stromal populations (**Supplementary Figure S3B**). In contrast, myeloid immunoregulatory markers were strongly enriched in microglia/macrophages and were also detected in neutrophils and, to a lesser extent, endothelial and stromal populations (**Supplementary Figure S3C**). All three immunoregulatory marker scores were significantly higher in mesenchymal tumors than in classical and proneural tumors (**Supplementary Figures S3D–F**), whereas none differed significantly according to EGFR amplification status (**Supplementary Figures S3G–I**). Together, these observations indicate that the immunoregulatory scores are associated with GBM transcriptional subtype, particularly the mesenchymal state, whereas no clear association with EGFR amplification was detected.

Collectively, these single-cell and immune-context analyses indicate that the three functional gene subsets within the shared vascular-hypoxic signature are detected across endothelial, perivascular and myeloid compartments.

### 3.3 Selected functional gene subsets are associated with mesenchymal GBM identity and adverse clinical outcome

To determine whether selected genes from the three functional gene subsets were reflected in established GBM molecular states, the most highly enriched genes from each subset were evaluated across classical, mesenchymal and proneural GBM subtypes. In the TCGA-GBM RNA-seq cohort, the most highly enriched angiogenesis and vascular remodeling genes, immune and inflammatory signaling genes, and shared immunovascular genes showed preferential expression in mesenchymal GBM compared with classical and proneural tumors (**Figures 4A–C**). Analysis of the same predefined genes in the independent CGGA cohort showed a broadly concordant mesenchymal-associated expression pattern, supporting cross-cohort consistency of the subtype association (**Supplementary Figure S4**).

**Figure 4:**
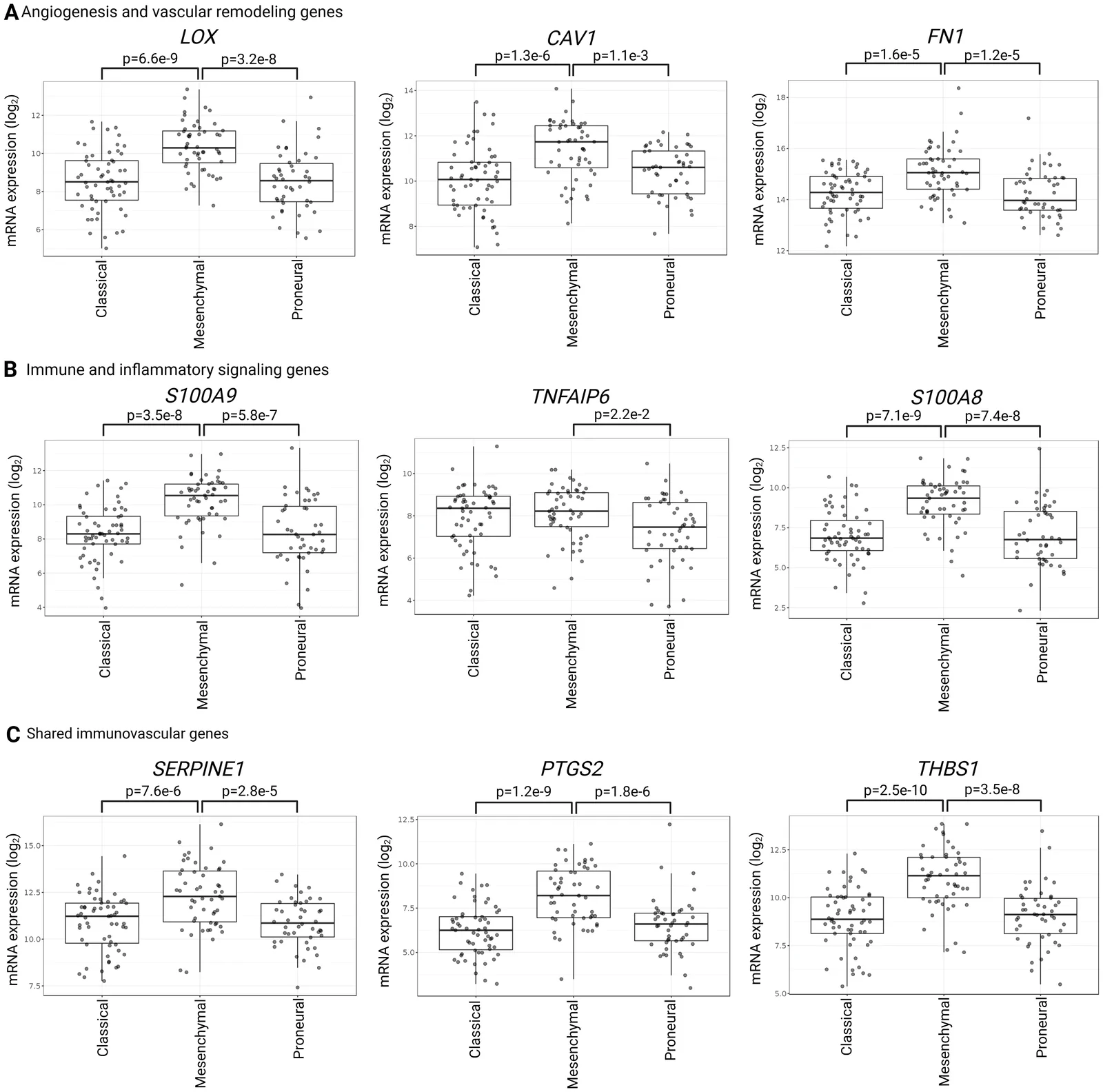
Top enriched genes from the three functional gene subsets are preferentially expressed in mesenchymal GBM. **(A)** Expression of the three most highly enriched genes from the angiogenesis and vascular remodeling subset (*LOX*, *CAV1*, and *FN1*) across classical, mesenchymal, and proneural GBM transcriptional subtypes in the TCGA-GBM RNA-seq cohort. **(B)** Expression of the three most highly enriched genes from the immune and inflammatory signaling subset (*S100A9*, *TNFAIP6*, and *S100A8*) across classical, mesenchymal, and proneural GBM transcriptional subtypes in the TCGA-GBM RNA-seq cohort. **(C)** Expression of the three most highly enriched genes from the shared immunovascular subset (*SERPINE1*, *PTGS2*, and *THBS1*) across classical, mesenchymal, and proneural GBM transcriptional subtypes in the TCGA-GBM RNA-seq cohort. For all panels, boxplots show log2-transformed mRNA expression values, with individual points representing tumor samples. Statistical significance is reported as exact adjusted *p*-values.

To assess clinical association, the three functional gene subsets were analyzed together as a predefined multigene set in SurvExpress. This analysis stratified patients into groups with distinct overall survival across TCGA-GBM and two independent GBM cohorts, Gravendeel GSE16011 and Lee/Nelson GSE13041. In all three cohorts, high-risk groups showed significantly shorter overall survival (**Supplementary Figure S5A-C**). Gene-level expression patterns across risk groups further indicated that adverse outcome was associated with coordinated elevation of multiple genes across the angiogenesis and vascular remodeling, immune and inflammatory signaling, and shared immunovascular subsets, rather than dominance of a single transcript (**Supplementary Figures S5A-C**; **Supplementary File 1**).

Subtype-stratified analysis showed the strongest adverse survival associations in mesenchymal GBM. High angiogenesis and vascular remodeling and shared immunovascular signature expression were associated with shorter overall survival (HR = 2.4, p = 0.0073 and HR = 1.9, p = 0.048, respectively), whereas the immune and inflammatory signaling subset showed a concordant but non-significant trend (HR = 1.7, p = 0.073; **Supplementary Figures S6A-C**). No clear survival separation was observed in classical or proneural tumors. These exploratory analyses therefore suggest that adverse survival associations of the vascular-hypoxic program are most evident in the mesenchymal transcriptional context.

## 4 Discussion

This study identifies a shared vascular-hypoxic transcriptional program in GBM that integrates vascular remodeling, inflammatory signaling, and immune-related features across two anatomically distinct tumor niches. Analysis of IvyGAP anatomical transcriptomes, combined with functional enrichment, single-cell mapping, immune-context analyses and survival modeling, revealed convergence of HBV/MVP and PNZ/PAN regions on a 95-gene program encompassing angiogenesis, extracellular-matrix and tissue remodeling, inflammatory signaling, chemotaxis and immune-cell trafficking. Rather than redefining histological niches that are increasingly resolved by spatial profiling, these findings delineate a common transcriptional axis linking vascular- and hypoxia/necrosis-associated compartments to endothelial/perivascular and myeloid populations, mesenchymal GBM identity, stromal and immune-exclusion-related features, and adverse clinical outcome across independent datasets.

Recent spatial studies place these findings within an increasingly detailed framework of GBM tissue organization. Greenwald et al. identified a multilayered spatial architecture associated with hypoxia, extending from hypoxic/necrotic regions toward the infiltrative tumor edge, and proposed hypoxia as a long-range organizer of cellular states in GBM (2). Liu et al. independently reported marked immunosuppression in perinecrotic regions and a more pro-inflammatory profile in perivascular regions (6). More recently, Sonpatki et al. generated a high-resolution multimodal atlas that refined IvyGAP-derived anatomical features and revealed overlapping vascular and immune signatures across HBV, MVP and PNZ. Immune diversity decreased further in hypoxic PNZ/PAN regions, where macrophages remained prominent (3). Wu et al. further resolved peri-necrotic hypoxic and inflammatory ecosystems, showing that these niches are spatially adjacent or partially overlapping and that hypoxic regions can serve as hubs of immune-checkpoint expression (26). The present analysis complements these spatial studies by identifying a transcriptional program shared across anatomically distinct vascular- and hypoxia/necrosis-associated compartments, without implying equivalence between these niches. This shared program provides a molecular link between classical histopathological structures and the multicellular microenvironments resolved by recent high-resolution spatial profiling.

Recent mechanistic work also supports reciprocal coupling between vascular cells and macrophages in GBM. Yang et al. identified a mesenchymal-like endothelial population that forms an immunosuppressive vascular niche through a TWIST1/SATB1/osteopontin program promoting immunosuppressive macrophage polarization and limiting T-cell activity (14). Conversely, Wang et al. identified a peri-necrotic hypoxia-associated monocyte-derived macrophage population that disrupts endothelial adherens junctions through adrenomedullin signaling, increases vascular permeability and can be therapeutically targeted to promote vascular normalization (12). Together, these studies support reciprocal endothelial-to-myeloid and myeloid-to-endothelial interactions within the GBM microenvironment. The single-cell re-analyses presented here are consistent with this framework: vascular-remodeling genes were concentrated in endothelial and perivascular populations, whereas immune/inflammatory genes were most prominent in myeloid cells but remained detectable in vascular-associated populations. Vascular-remodeling genes were also positively associated with macrophage and monocyte abundance and with immunoregulatory markers. These findings extend recent mechanistic observations by placing vascular- and myeloid-associated transcriptional features within an anatomically anchored program shared across vascular- and hypoxia/necrosis-associated GBM compartments and supported across independent datasets. Importantly, these associations define a multicellular transcriptional context rather than a specific causal pathway or direction of signaling. Consistent with this multicellular organization, checkpoint/T-cell dysfunction markers localized predominantly to microglia/macrophages and T cells, while all three immunoregulatory signatures were enriched in mesenchymal GBM but showed no clear association with EGFR amplification. These findings associate the immunoregulatory context with the mesenchymal transcriptional state, while no clear association with EGFR amplification was detected.

Stromal remodeling may represent an additional link between vascular pathology and immune exclusion. In matched primary and recurrent GBM, Onubogu et al. described collagen-rich perivascular extracellular matrix structures containing fibroblast-like cells and immunosuppressive macrophages, with an organization that could restrict access of other immune populations to the tumor parenchyma (15). Sonpatki et al. similarly identified immune-glial and immune-fibrotic niches characterized by vascular, fibroblast and immune components and associated with immune suppression and tissue-remodeling programs (3). A recent computational study further described a DAM-like macrophage signature centered on MMP9, MMP14 and CCL2 that was associated with stromal remodeling, T-cell restriction and adverse outcome in GBM (27). In our data, the shared vascular-hypoxic signature was enriched for focal adhesion, ECM-receptor interaction, wound-healing and coagulation-related pathways, while vascular-remodeling genes showed positive associations with stromal and exclusion scores and with immunoregulatory markers.

The translational implications of these findings should be considered in the context of clinical experience with immune checkpoint blockade and antiangiogenic therapy in GBM. Although vascular normalization provides a strong biological rationale for combination strategies and preclinical studies indicate that VEGF blockade can enhance immune-cell delivery and activity (28, 29), immune checkpoint inhibitors have shown limited efficacy in unselected GBM populations. In recurrent GBM, the phase III CheckMate 143 trial showed that nivolumab did not improve overall survival compared with bevacizumab (30). In newly diagnosed disease, CheckMate 498 did not demonstrate benefit from nivolumab plus radiotherapy over temozolomide plus radiotherapy in patients with unmethylated MGMT promoter, and CheckMate 548 showed no survival benefit from adding nivolumab to radiotherapy and temozolomide in patients with methylated or indeterminate MGMT promoter (31, 32). In recurrent GBM, randomized phase II evaluation of pembrolizumab with bevacizumab versus pembrolizumab alone demonstrated limited activity without a clear survival advantage (33), while durvalumab alone or combined with standard- or reduced-dose bevacizumab did not meet predefined efficacy endpoints (34). More recently, a phase II study of pembrolizumab plus re-irradiation with bevacizumab continuation in bevacizumab-refractory GBM met a historical OS-6 benchmark, whereas survival was not improved in the bevacizumab-naive cohort; the authors emphasized the need for randomized confirmation (35). Collectively, these studies suggest that vascular targeting may modulate the immune microenvironment but is not sufficient to overcome GBM immune resistance in broadly selected patients. The vascular-hypoxic program described here should therefore not be interpreted as a treatment-predictive biomarker, but it provides a candidate framework for future studies testing whether immunovascular and stromal states can stratify response to vascular-immune combination strategies.

Several limitations should be considered. This study is based on publicly available transcriptomic datasets, and the analyses are shaped by the resolution, annotations and sample composition of each platform. Although IvyGAP provides a unique anatomical framework for GBM, additional spatial transcriptomic, multiplex tissue-based and functional studies will be needed to resolve patient-specific cellular interactions and define how endothelial, perivascular, tumor and myeloid populations interact within vascular-hypoxic niches. An additional limitation is the absence of an institutional validation cohort with matched tumor material, molecular annotation and clinical follow-up; consequently, the clinical and microenvironmental associations identified here require validation in independent, clinically annotated tissue cohorts.

Nevertheless, the convergence of anatomical, functional, single-cell, immune-context, subtype and survival analyses supports a model in which vascular and hypoxia/necrosis-associated GBM niches share an immunovascular remodeling state. By linking angiogenesis and vascular dysfunction with hypoxic stress, stromal remodeling, myeloid immunoregulation and exclusion-associated features, this framework complements recent spatial and mechanistic studies and defines a testable program for future spatial validation and functional perturbation in clinically annotated GBM samples.

## Data Availability

The original contributions presented in the study are included in the article/**Supplementary Material**. Further inquiries can be directed to the corresponding author.
