# Supplementary material for: A shared vascular-hypoxic transcriptional program is associated with immune organization in glioblastoma
Source: Front Immunol. 2026 Oct 28;17:1960638. doi: 10.3389/fimmu.2026.1960638 (PMC13752119; doi:10.3389/fimmu.2026.1960638)
Supplement: Supplementary file 1 [file SupplementaryFile1.docx]

**Figure S1. Vascular and hypoxia-associated IvyGAP programs define complementary enrichment patterns and shared extracellular matrix-immune network modules.**

(A) Gene Ontology Biological Process enrichment analysis in vascular-associated regions (HBV+MVP) compared with reference tumor compartments (LE+IT+CT). The x-axis shows fold enrichment. Dot size indicates the number of genes assigned to each term, and color indicates enrichment significance expressed as -log_10_(FDR).
(B) Gene Ontology Biological Process enrichment analysis of genes enriched in hypoxia/necrosis-associated IvyGAP regions (PNZ+PAN), compared with the same reference tumor compartments (LE+IT+CT). The x-axis shows fold enrichment. Dot size indicates the number of genes assigned to each term, and color indicates enrichment significance expressed as -log_10_(FDR).
(C) STRING protein functional association network generated from the 95-gene shared vascular-hypoxic signature. Nodes represent proteins encoded by genes in the shared signature, and edges represent STRING functional associations. Node colors indicate network clusters or functional annotation groups shown in the legend.

**Table S1. Functional gene subsets selected from the shared vascular–hypoxic signature.**

| Functional gene subset | Assigned genes |
| --- | --- |
| Angiogenesis and vascular remodeling genes | *LOX, CAV1, FN1, TGFBI, ITGA5, LOXL2, ADAMTS1, NRP1, APLN, ANXA2, RUNX1, BTG1, EPAS1* |
| Immune and inflammatory signaling genes | *S100A9, TNFAIP6, S100A8, DUSP1, ISG20, FCGR2B, CCL3, LYZ, CXCL1, GPR183, LY96, LGALS1, FAS, CXCL6* |
| Shared immunovascular genes | *SERPINE1, PTGS2, THBS1, CXCR4, SOCS3, TNFAIP3, CCL8, JUNB, C5AR1, B4GALT1, ANXA1* |

**Figure S2. Single-cell mapping and graphical scoring support endothelial, perivascular, myeloid and tumor compartment enrichment of IvyGAP-derived vascular-hypoxic niche gene programs in GBM.**

**(A, C, E)** UMAP visualization of the three predefined gene subsets in an independent brain vascular single-cell reference dataset: angiogenesis and vascular remodeling genes (**A**), immune and inflammatory signaling genes (**C**), and shared immunovascular genes (**E**).

**(B, D, F)** Dot-plot summaries of the same gene subsets across annotated cell populations: angiogenesis and vascular remodeling genes (**B**), immune and inflammatory signaling genes (**D**), and shared immunovascular genes (**F**).

Dot color represents average expression, whereas dot size indicates the fraction of cells with detectable expression within each population.

**Figure S3. Cellular distribution and molecular associations of immunoregulatory marker signatures in GBM.**

(**A–C**) Single-cell expression of predefined checkpoint/T-cell dysfunction markers (**A**), immunosuppressive cytokine signaling markers (**B**), and myeloid immunoregulatory markers (**C**) across major cell populations in the GBM microenvironment. Dot size represents the proportion of cells with detectable expression, and color intensity indicates average expression. (**D–F**) Comparison of checkpoint/T-cell dysfunction (**D**), immunosuppressive cytokine signaling (**E**), and myeloid immunoregulatory (**F**) signature scores across classical, mesenchymal, and proneural GBM transcriptional subtypes.

(**G–I**) Comparison of checkpoint/T-cell dysfunction (**G**), immunosuppressive cytokine signaling (**H**), and myeloid immunoregulatory (**I**) signature scores between EGFR-amplified (EGFR_AMP) and non-amplified (EGFR_NO_AMP) GBM tumors.

Statistical significance is indicated by adjusted p-values.

**Figure S4. Independent CGGA analysis supports Mesenchymal enrichment of representative genes from the three vascular-hypoxic functional subsets.**

Expression of representative genes from the predefined IvyGAP-derived vascular-hypoxic signature was evaluated across classical, mesenchymal, and proneural GBM transcriptional subtypes in the CGGA validation cohort.

(**A**) Expression of genes from the angiogenesis and vascular remodeling subset (*LOX*, *CAV1*, and *FN1*) across GBM transcriptional subtypes.

(**B**) Expression of genes from the immune and inflammatory signaling subset (*S100A9*, *TNFAIP6*, and *S100A8)* across GBM transcriptional subtypes.

(**C**) Expression of genes from the shared immunovascular subset (*SERPINE1*, *PTGS2*, and *THBS1*) across GBM transcriptional subtypes.

For all panels, boxplots show log2-transformed mRNA expression values, with individual points representing tumor samples. Statistical significance is reported as exact adjusted
*p*-values.

**Figure S5. Cross-cohort survival analyses associate the IvyGAP-derived vascular–hypoxic program with overall survival in GBM.**

The predefined IvyGAP-derived vascular-hypoxic signature, comprising the angiogenesis and vascular remodeling, immune and inflammatory signaling, and shared immunovascular gene subsets, was evaluated in independent GBM cohorts using SurvExpress-based risk stratification.

(**A**) TCGA-GBM cohort. Left panel: overall survival curves for patients classified into low-risk and high-risk groups. Right panel: expression of individual genes from the three functional subsets in low-risk and high-risk tumors.

(**B**) Gravendeel French Glioblastoma GSE16011 cohort. Left panel: overall survival curves for patients classified into low-risk and high-risk groups. Right panel: expression of individual genes from the three functional subsets across the two risk groups.

(**C**) Lee Nelson Glioblastoma GSE13041 GPL96 cohort. Left panel: overall survival curves for patients classified into low-risk and high-risk groups. Right panel: expression of individual genes from the three functional subsets across the two risk groups.

Hazard ratios, confidence intervals, and platform-derived log-rank p-values are shown for each survival comparison. Gene-level boxplots display platform-derived unadjusted p-values for differences between risk groups.

**Figure S6. Subtype-stratified overall survival analysis of the three functional gene subsets in GBM.**

Kaplan-Meier overall survival analyses were performed separately in classical, mesenchymal and proneural GBM transcriptional subtypes using predefined gene signatures representing the three functional subsets: **(A)** Angiogenesis and vascular remodeling gene subset. **(B)** Immune and inflammatory signaling gene subset. **(C)** Shared immunovascular gene subset. Within each transcriptional subtype, patients were stratified into low- and high-expression signature groups. Hazard ratios (HRs), log-rank *p*-values and group sizes are shown for each comparison.
